# PreTP-2L: identification of therapeutic peptides and their types using two-layer ensemble learning framework

**DOI:** 10.1093/bioinformatics/btad125

**Published:** 2023-04-03

**Authors:** Ke Yan, Yichen Guo, Bin Liu

**Affiliations:** School of Computer Science and Technology, Beijing Institute of Technology, Beijing 100081, China; School of Computer Science and Technology, Beijing Institute of Technology, Beijing 100081, China; School of Computer Science and Technology, Beijing Institute of Technology, Beijing 100081, China; Advanced Research Institute of Multidisciplinary Science, Beijing Institute of Technology, Beijing 100081, China

## Abstract

**Motivation:**

Therapeutic peptides play an important role in immune regulation. Recently various therapeutic peptides have been used in the field of medical research, and have great potential in the design of therapeutic schedules. Therefore, it is essential to utilize the computational methods to predict the therapeutic peptides. However, the therapeutic peptides cannot be accurately predicted by the existing predictors. Furthermore, chaotic datasets are also an important obstacle of the development of this important field. Therefore, it is still challenging to develop a multi-classification model for identification of therapeutic peptides and their types.

**Results:**

In this work, we constructed a general therapeutic peptide dataset. An ensemble-learning method named PreTP-2L was developed for predicting various therapeutic peptide types. PreTP-2L consists of two layers. The first layer predicts whether a peptide sequence belongs to therapeutic peptide, and the second layer predicts if a therapeutic peptide belongs to a particular species.

**Availability and implementation:**

A user-friendly webserver PreTP-2L can be accessed at http://bliulab.net/PreTP-2L.

## 1 Introduction

Peptides are in a balanced state in the human body, and play a crucial role in human physiology (Vazquez-Prieto *et al.*, 2017; Vázquez-Prieto *et al.*, 2017). In addition, some therapeutic peptides play an important role in the field of biopharmaceutics. Recently, various therapeutic peptides have been used in the field of medical research, and have great potential in the design of therapeutic schedules (Yan *et al.*, 2022a). Therefore, developing data-driven computational methods are essential for the therapeutic peptide prediction (Charoenkwan *et al.*, 2021; Yan *et al.*, 2022a).

There are various types of peptides, and each of them has particular characteristics and roles. For example, anti-cancer peptides (ACP) have been utilized as one of the potential treatments in curing cancer (Borghouts *et al.*, 2005; Yan *et al.*, 2022b). Anti-angiogenic peptides (AAP) develop the therapeutic treatments for angiogenesis disease (Gupta *et al.*, 2017). Drug delivery vehicle (DDV) can be utilized to enhance the delivery of drug molecules (Hasan *et al.*, 2021; Vargason *et al.*, 2021).

In the last decade, prediction therapeutic peptides based on machine-learning methods have been widely used in computational biology (Guo *et al.*, 2021). Different methods are trained and evaluated with different datasets (Wei *et al.*, 2018; Rao *et al.*, 2020; Agrawal *et al.*, 2021). As a result, the datasets used in the current studies are particularly heterogeneous.

There are two important parts in the existing computational models, including feature extraction and machine-learning classifier construction. For feature extraction, several excellent efforts have been captured to describe the peptides in the literature. For example, amino acid composition and dipeptide composition are widely used features for representing the composition of the peptide sequences (Qiang *et al.*, 2018). Furthermore, pseudo amino acid composition (Pse-AAC) was proposed to extract sequence information (Shen and Chou, 2008). The feature representation methods mentioned above are specifically proposed for peptides. In order to extract the discriminative features for specific peptides, various peptides prediction models utilize the SFS and mRMD to extract the specific features, such as PEPred-Suite (Wei *et al.*, 2019) and PPTPP (Zhang and Zou, 2020). For machine-learning classifier construction, various predictors are developed to distinguish true therapeutic peptides. For example, ACPredStackL (Ison *et al.*, 2016) utilizes the stacking framework, which combines the prediction information from several basic predictors. Furthermore, PPTPP (Zhang and Zou, 2020) and PEPred-Suite (Wei *et al.*, 2019) are two widely used therapeutic peptides predictors based on random forest (RF) (Breiman, 2001). As far as we known, most of the previous predictor are focused on the single functional therapeutic peptide recognition.

However, with the number of multiple-function therapeutic peptides increasing, there is no multi-classification predictor that can accurately predict all therapeutic peptide types due to the different types of peptides with various characteristics. Therefore, we proposed a general predictor PreTP-2L based on two-layer framework for therapeutic peptide recognition. The first layer predicts whether a peptide sequence is a therapeutic peptide, and the second layer predicts if a therapeutic peptide belongs to a particular function. The first layer is based on convolutional neural network (CNN) (O'Shea and Nash, 2015) for training, while the second layer utilizes VGG13 (Sengupta *et al.*, 2019) network. PreTP-2L utilizes a generic predictor to learn the discriminative information among the different therapeutic peptides simultaneously. The proposed method utilizes the deep-learning network CNN and VGG13 frameworks to optimize the feature from the peptide sequence. Furthermore, we constructed a comprehensive dataset by collecting the comprehensive range of therapeutic peptides. The dataset contains 13 604 sequences from 16 major functions. To distinguish therapeutic peptides from non-therapeutic peptides, we extracted, and constructed a negative sample dataset with 11 230 sequences from Uniprot (Bairoch *et al.*, 2005). For convenience, we constructed a webserver of PreTP-2L to predict unknown peptide sequences, which can be accessed at http://bliulab.net/PreTP-2L.

## 2 Materials and methods

### 2.1 Benchmark dataset

**Figure 1:**
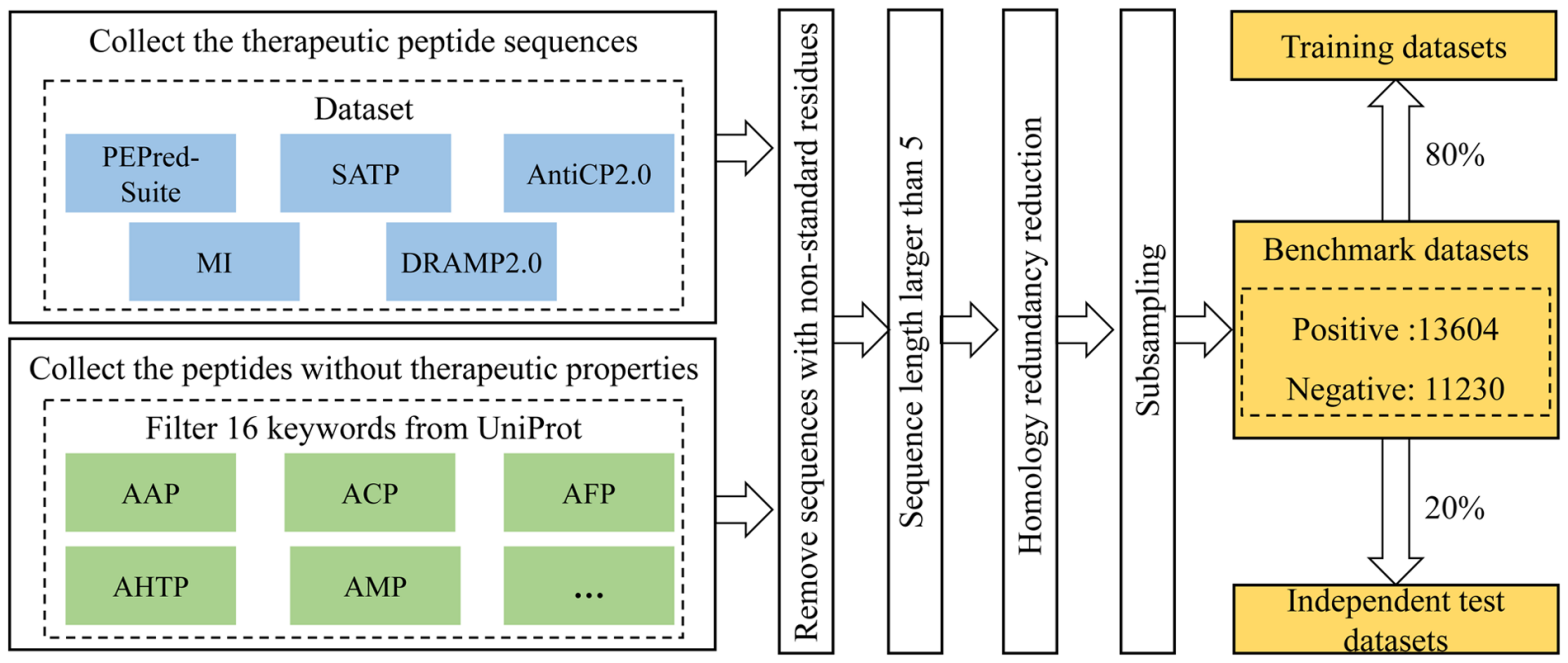
The flowchart for constructing the training datasets and independent test datasets

In this study, we utilized 16 various therapeutic peptide datasets, including AAP (Ettayapuram Ramaprasad *et al.*, 2015), Anti-Bacterial Peptides (ABP) (Lata *et al.*, 2007), ACP (Wei *et al.*, 2018), Anti-Fungal Peptides (AFP) (Singh *et al.*, 2016), Anti-Hypertensive Peptides (AHTP) (Basith *et al.*, 2020), Anti-Inflammatory Peptides (AIP) (Manavalan *et al.*, 2018), Anti-Microbial Peptides (AMP), Anti-Parasitic Peptides (APP) (Singh *et al.*, 2016), Anti-Tubercular Peptides (ATbP) (Basith *et al.*, 2020), Anti-Viral Peptides (AVP) (Thakur *et al.*, 2012), Cell–cell Communication Peptides (CCC) (Singh *et al.*, 2016), Cell-penetrating peptides (CPP) (Wei *et al.*, 2017), DDV (Singh *et al.*, 2016), Polystyrene surface Binding peptides (PBP) (Li *et al.*, 2017), Quorun Sensing Peptides (QSP) (Rajput *et al.*, 2015), and Toxic Peptides (TXP) (Singh *et al.*, 2016). The statistical details of these datasets are shown in Table 1. The flowchart for constructing the benchmark dataset is illustrated in Fig. 1.

**Table 1.** Summary of datasets.

Functional category	Training datasets	Independent test datasets
AAP	109	26
ABP	888	222
ACP	585	144
AFP	657	164
AHTP	673	168
AIP	1388	346
AMP	1760	438
APP	137	34
ATbP	17	4
AVP	1499	374
CCC	236	58
CPP	481	118
DDV	697	172
PBP	84	20
QSP	113	26
TXP	1574	392

In this study, the positive peptides were collection of therapeutically peptides from different latest research literature, including the PEPred-Suite (Wei *et al.*, 2019), SATP (Singh *et al.*, 2016), AntiCP2.0 (Agrawal *et al.*, 2021), MI (Basith *et al.*, 2020), and DRAMP2.0 (Kang *et al.*, 2019). The negative peptide sequences are extracted from Uniprot (Wei *et al.*, 2019).

For positive samples, different literature reviews are integrated to integrate various peptide types and datasets. As for some common peptide types, there are many relevant studies, and we integrated and filtered directly from the previous datasets. For instance, AFP, CVP, and TXP are extracted from SATPdb (Singh *et al.*, 2016). We selected the positive sequence with length larger than five and eliminated the non-standard residues. Finally, we removed the redundant sequences by using CD-HIT (Li and Godzik, 2006) with a cut-off of 80% (Basith *et al.*, 2020).

In the process of constructing the negative samples of the first layer, we extracted the negative samples. The negative dataset was constructed from Uniprot (Wei *et al.*, 2019) by filtering sequences related 16 therapeutic peptide functions, including AAP, ABP, ACP, AFP, AHTP, AIP, AMP, APP, ATbP, AVP, CCC, CPP, DDV, PBP, QSP, and TXP. We eliminated the non-standard residues and selected negative samples with lengths between 5 and 100 residues to maintain the same distribution of lengths as the positive samples. Then, we removed the redundant sequences sharing >40 sequence similarity (Pirtskhalava *et al.*, 2021) with any positive samples by using BlastClust (Dondoshansky and Wolf, 2002). In order to make the length distributions of the positive and negative sequences the same, we filtered out longer negative samples and subsampled 11 230 negative samples.

As for both positive datasets and negative datasets, we divided the different peptide types according to the ratio 8:2 as the training dataset and test dataset, respectively (Basith *et al.*, 2020).

### 2.2 Overview of PreTP-2L

**Figure 2:**
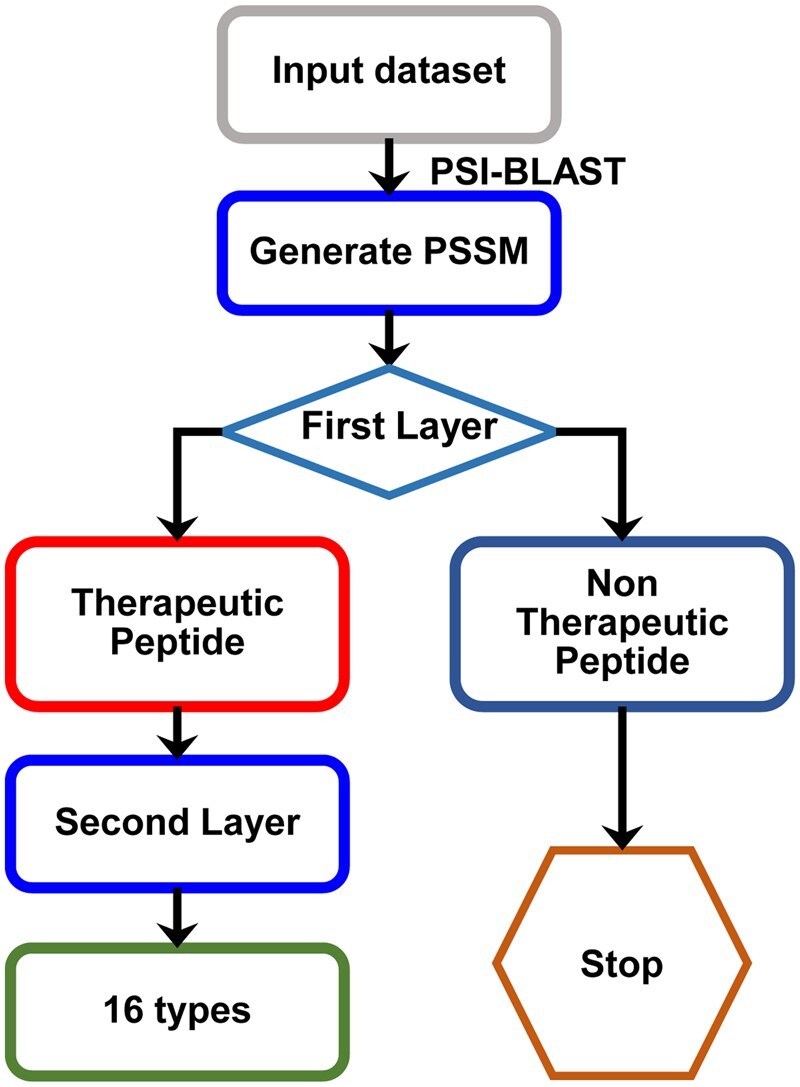
The flowchart of PreTP-2L. It contains of three main steps: (i) feature extraction, (ii) first layer, and (iii) second layer

The framework of PreTP-2L is shown in Fig. 2. The model consists of two layers. The first layer model predicts whether it belongs to therapeutic peptide, and the second layer model predicts its type. Both the two layers use position-specific scoring matrix (PSSM) and convolution neural network. The first layer uses CNN to relearn features through convolution and average pooling. CNN extracted the evolutionary information through the feature mapping framework. The second layer uses VGG13 to add more convolution layers to extract specific features, which is related to the multi-functional therapeutic peptides.

### 2.3 Feature extraction methods

Feature extraction is an important step for constructing a method for predicting therapeutic peptides. Each sequence P is represented as (Li *et al.*, 2021):


(1) $$P=R_{1}R_{2}R_{3}R_{4}R_{5}R_{6}R_{7}\ldots R_{L}.$$

In this experiment, we utilize the PSSM (Altschul *et al.*, 1997) to represent peptide sequences with a cut-off of 100 residues and 50 residues for the first layer and the second layer in PreTP-2L, respectively. For a protein length shorter than the cut-off, we utilize the zero padding strategy (Muquet *et al.*, 2002). PSSM is used to extract the evolutionary sequence characteristics of proteins, and it can briefly and unambiguously indicate the characteristics of proteins (Holm and Sander, 1998; Liu and Li, 2019; Liu *et al.*, 2020). For the input therapeutic peptide sequences, the corresponding PSSM can be generated through PSI-BLAST searching against the non-redundant database nrdb90 with iterations = 3 and *e*-value = 0.05. Since some peptide sequences may be short, their PSSM cannot be generated through PSI-BLAST, and the blosum62 (Henikoff and Henikoff, 1992) was used instead of the PSSM. The PSSM can be represented as: where $S_{i,\;j}\left(i\in \left[1,L\right],j\in \left[1,20\right]\right)$ represents the score of standard amino acid j in the position of i of peptide sample, and L is peptide sequence length.


(2) $$\mathrm{PSSM}=\;\left[\begin{array}{ccc} S_{1,1} & \cdots & S_{1,20}\\ \vdots & \ddots & \vdots \\ S_{L,1} & \cdots & S_{L,20} \end{array}\right],$$

### 2.4 First layer network

**Figure 3:**
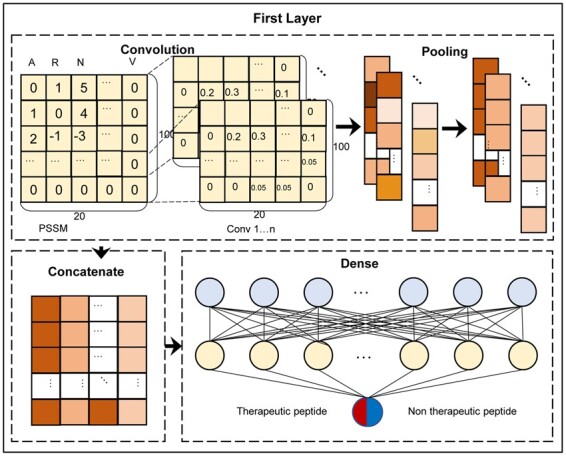
Framework of the first layer in PreTP-2L. There are four main steps: (i) convolution, (ii) pooling, (iii) concatenate, and (iv) dense

The flowchart of the first layer of PreTP-2L is shown in Fig. 3. In order to better extract useful features from PSSM, we constructed a multiscale CNN (Zhang *et al.*, 2020). The whole CNN contains four parts. In each part, it includes convolutional layers (Burrus and Parks, 1985) followed by Rectified Linear Unit (Relu) and a max pooling layers (Tolias *et al.*, 2015). In this experiment, the multiscale CNN consists of four kernel sizes (5, 7, 10, and 20). The convolutions can detect known peptide sequence features, and learn potential evolutionary peptide sequence patterns. The four convolutions are complementary, strengthening the robustness of CNN. The outputs of convolutional layer represent scanning data of correlated convolution kernels. One convolution kernel scanning result indicates the probability that the corresponding pattern arising at specific position of the peptide sequence. Kernel scanning results of convolution kernel *k* at position *i* are represented as (Alzubaidi *et al.*, 2021): where *l* indicates kernel size, $w_{j,c}^{k}$ represents the weight of row *j* and column *c* in kernel *k*, $s_{j+i,c}$ is the element in row (*j *+* i*) and column *c* of PSSM, and $b_{k}$ is the *k*th bias of convolutional kernel.


(3) $$\mathrm{SC}\left(k,i\right)=\;b_{k}+\sum_{j=1}^{l}\sum_{c=1}^{20}w_{j,c}^{k}\times s_{j+i,c},$$

The max pooling layer filtrates the convolution kernel scanning results. In the further process, the maximum value of each convolution kernel scanning results was selected through max pooling layer. The values above are pattern features representing the corresponding data appearing in the peptide sequence. The larger the value is, the more likely the corresponding pattern appears in the peptide sequence. As for concatenate and flatten layer, pooling features of each peptide sequence were concatenated and flattened for subsequent layer. Then, the fully connected layer consists of ReLU (Agarap, 2018) as activation function. This layer plays an important role in learning the characteristics of different kinds of proteins. The outputs of the concatenate and flatten layer indicates the associations of convolution kernels in various types of peptides. In the output layer, the features mentioned above can predict whether an input sequence is a therapeutic peptide or not. This layer includes two neurons and two outputs. The neurons outputs represent the probabilities of the sequence being a therapeutic peptides or not, which can be calculated through softmax (Jang *et al.*, 2016) function. The dropout was used after fully connected layer to reduce the possibility of over-fitting. Furthermore, the binary cross entropy function was used to calculate loss, which can be calculated as (Zhang and Sabuncu, 2018): where θ are the training parameters, X are the inputs, and y are the true outputs. $y_{i}\left(x\right)$ represents the expected outputs of x for *j*-th label, $p_{j}\left(x\right)$ represents the predicted probabilities of x for label *j*, and w is positive sequences weight in order to reduce noise from cluttered samples. In that case, Ω(θ) calculates the *L*2 regularization, which can suppress the problem of over-fitting, which is calculated through Zhang and Sabuncu (2018): where *n* is the scale of training parameters.


(4) $$J\left(\theta ,\;X,\;y\right)=\;-\sum_{x}^{X}\sum_{j=1}^{2}\left[w\times y_{i}\left(x\right)\log\left(p_{j}\left(x\right)\right)+\left(1-y_{j}\left(x\right)\right)\log\left(1-p_{j}\left(x\right)\right)\right]+\Omega \left(\theta \right),$$

(5) $$\Omega \left(\theta \right)=\;\frac{1}{2}\sum_{i=1}^{n}{\left|\theta_{i}\right|}^{2},$$

After the first layer of the network, predicted therapeutic peptides will be fed into the second layer of the network to predict therapeutic peptide types.

### 2.5 Second layer network

**Figure 4:**
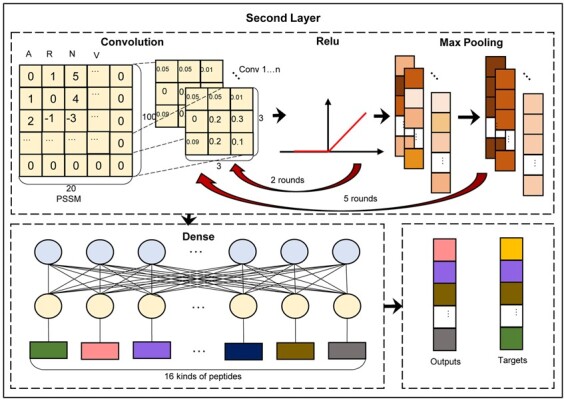
Framework of the second layer in PreTP-2L. There are four main steps: (i) convolution, (ii) ReLU, (iii) max pooling, and (iv) dense

The framework of the second layer in PreTP-2L is shown in Fig. 4. The optimized convolution neural network Visual Geometry Group (VGG13) (Simonyan and Zisserman, 2014) was utilized to train the multi-classification datasets. In order to extract the evolutionary features from PSSM, we introduced VGG13 to train the second layer model consisting of repeated convolution and pooling operation. The whole CNN contains feature part and classification part. In the feature part, it contains five segments, and each segment includes two cycles of convolution and ReLU. The tail of each segment is pooled to reduce the data size. The convolutions can detect known peptide sequence features, and learn sequence patterns. Each block is interconnected with each other. The max pooling part of last block and the convolution part of the next block is connected with each other.

A great progress of VGG is to mimic the effect of larger receptive fields, such as 5 × 5 and 7 × 7, by sequentially employing multiple 3 × 3 convolutions. The VGG13 utilizes all convolutional kernels (3 × 3) and pooling kernels (2 × 2). Then, it keeps enrich the structure of network to increase the performance. The deeper the network, the number of parameters does not grow much, and the number of parameters is mainly in the three fully connected layers. The number of convolution kernels within each segment is the same. The convolution kernels are varied from 64, 128, 256 to 512.

Between classifier segment and feature segment, there is an average pool used to filter the results. Average pooling contributes to the reduction of parameter dimensionality and the complete transfer of information (Boureau *et al.*, 2010) in a very large and representative model. In the classifier block, correlation features are used to predict the therapeutic peptide types. This block consists of three linear modules and two dropout layers after ReLU. The dropout operation is used after the ReLU layer to decrease the possibility of over-fitting. In the network, weighted binary cross entropy function was used to calculate loss, which can be calculated as (Ho and Wookey, 2019): where *N* is the number of samples, ${\hat{y}}^{i}$ represents probability of sample *i*, and $y^{i}$ is the label of sample *i*. The Adadelta (Zeiler, 2012) method is used as optimization algorithm. The learning rate of Adadelta will grow with the inverse of the gradient, which indicates that a higher gradient leads to a smaller learning rate, and *vice versa*. In this case, Adadelta can solve the problem of constant learning rate in the ordinary Stochastic Gradient Descent (SGD) (Cherry *et al.*, 1998) method. Finally, the second layer outputs 16 predicted scores for being different peptide types.


(6) $$L=\sum_{i=1}^{N}y^{i}l\mathrm{og}{\hat{y}}^{i}+\left(1\;–\;y^{i}\right)\log\left(1-\;{\hat{y}}^{i}\right),$$

In the training process of VGG13 network, we export the pre-training model from torchvision (Albardi *et al.*, 2021), and use training dataset mentioned in Section 2.1 to fine tune. Finally, we evaluate the performance of the proposed method on the independent test set.

### 2.6 Performance evaluation

For the first layer in PreTP-2L, we use area under curve (AUC), accuracy (ACC), precision (SN+), specificity (SP), and Matthews correlation coefficient (MCC) to evaluate its performance (Basith *et al.*, 2021, 2022; Hasan *et al.*, 2022). AUC represents the area under the receiver operating characteristic curve. ACC is the percentage of correct samples predicted by the classifier in the total samples. SN+ and SP represent the possibility that the test positive sequences and the test negative sequences can be correctly predicted, respectively. In this experiment, SN+ represents the proportion of the TP out of the predicted positive sequences. SN+ is a very important evaluation index, which can be used to measure the ACC of the prediction for positive samples. MCC measures the correlation between real value and predicted value. MCC value close to one indicates that the prediction is very accurate while the value close to zero means that the prediction is not better than random guess, and the value close to −1 means that the prediction is seriously inconsistent with the real category. These performance measures can be calculated as (Powers, 2008;  Yan *et al.*, 2023): where TP is true positive, TN is true negative, FP is false positive, and FN is false negative.


(7) $$\mathrm{ACC}=\frac{\mathrm{TP}+\mathrm{TN}}{\mathrm{TP}+\mathrm{TN}+\mathrm{FN}+\mathrm{FP}},$$

(8) $$\mathrm{MCC}=\frac{\mathrm{TP}\times \mathrm{TN}-\mathrm{FP}\times \mathrm{FN}}{\sqrt{\left(\mathrm{TP}+\mathrm{FN})(\mathrm{TP}+\mathrm{FP})(\mathrm{TN}+\mathrm{FP})(\mathrm{TN}+\mathrm{FN}\right)}},$$

(9) $$\mathrm{SP}=\frac{\mathrm{TN}}{\mathrm{TN}+\mathrm{FP}},$$

(10) $$\mathrm{SN}+=\frac{\mathrm{TP}}{\mathrm{TP}+\mathrm{FP}},$$

## 3 Results and discussion

### 3.1 Performance of first layer and second layer in PreTP-2L

The first layer in PreTP-2L predicts whether the input peptide sequence is a therapeutic peptide or not, and the second layer in PreTP-2L is to predict the therapeutic peptide types. Its results on the independent test dataset are listed in Tables 2 and 3, from which we can see that PreTP-2L is able to accurately predict the therapeutic peptides and their types. Therefore, PreTP-2L method is able to accurately predict the therapeutic peptides.

**Table 2.** Results of first layer in PreTP-2L on the independent test dataset.

	AUC	ACC	SN	SP	MCC
PreTP-2L	0.955	0.912	0.928	0.891	0.821

**Table 3.** Results of second layer in PreTP-2L on the independent test dataset.

Type	ACC	SN+	SP	MCC
AAP	0.984	0.219	0.991	0.235
ABP	0.871	0.190	0.933	0.113
ACP	0.925	0.303	0.962	0.232
AFP	0.898	0.154	0.946	0.099
AHTP	0.962	0.690	0.974	0.705
AIP	0.890	0.568	0.925	0.534
AMP	0.766	0.283	0.859	0.146
APP	0.982	0.208	0.993	0.166
ATbP	0.753	0.346	0.957	0.226
AVP	0.826	0.376	0.899	0.273
CCC	0.976	0.431	0.989	0.392
CPP	0.943	0.388	0.962	0.422
DDV	0.928	0.488	0.964	0.481
PBP	0.992	0.444	0.996	0.418
QSP	0.992	0.463	0.993	0.469
TXP	0.912	0.684	0.942	0.656

### 3.2 VGG13 network can improve the prediction performance

In this section, we investigate the performance of the VGG13 neural network in the second layer of PreTP-2L. We compared the performance of VGG13 with some other CNNs, such as Resnet18 and Alexnet. The results of the experiments are shown in Table 4, indicating that the VGG13 framework is better than the other methods. Due to the information in the pooling layer loses, we take full advantage of the evolutionary information by increasing the channels. The number of the channels is set to 512. Meanwhile, the smaller convolution kernel with stacking can reduce the number of parameters. The reasons are that the multiple convolution layers in VGG13 with fewer parameters are more non-linear modules, making the CNN more suitable for feature learning.

**Table 4.** Comparison between VGG13 and the other convolution neural networks on independent test datasets.

Models	VGG13	Alexnet	Resnet18
Epoch	500	500	500
Loss	CrossEntropyLoss	CrossEntropyLoss	CrossEntropyLoss
Optimizer	Adadelta	Adadelta	Adadelta
ACC	0.45	0.41	0.40

We also compared the performance of the VGG13 network with different parameters. As shown in Supplementary Table S1, the first column shows the final hyperparameter data used by the model, while the second, third, and fourth columns are the results on the independent test dataset with modified training batch size, epochs, and optimize methods, respectively. The set of parameters used in our final model gives the best performance on the independent test dataset. The size of epoch determines the number of training iterations. In terms of optimization methods, SGD has the same learning rate for all parameters and easily converges to a local optimum. Meanwhile, Adadelta introduces an adaptive learning rate, which adjusts the learning rate at different times according to the training situation.

### 3.3 Comparing PSSM with the other feature extraction methods

In this section, we compared the performance of the evolutionary information features based on the PSSM with the handcrafted features. We utilize several existing sequence-based features for peptide feature representation. Kmer (Gao *et al.*, 2019) indicates the occurrence information of *k* adjacent amino acids (*k* ∈ [1, … , 5]). DP (Liu *et al.*, 2014a) is the Pse-AAC of distance-pairs and the reduced alphabet scheme containing sequence secondary structure and physicochemical property information. DT (Liu *et al.*, 2014b) combines evolutionary information from Top-n-gram and distance information between pairs of amino acids. DT utilizes the occurrence times of all possible Top-n-gram pairs to calculate the feature vector at a given distance.

Then, we evaluate the performance of the combination features of the Kmer, DP, and DT (denoted as KDD) with the PSSM features on the independent test dataset. The results are shown in Table 5, from which we can see that the PreTP-2L based on the evolutionary information PSSM outperforms the method with the sequence information KDD in terms of the ACC. Therefore, the proposed method using the evolutionary information PSSM captures the more discriminative features, indicating that the proposed method is an effective method for therapeutic peptide recognition.

**Table 5.** Comparison between different features on the independent test datasets.

Feature	PSSM	KDD
ACC	0.45	0.26

### 3.4 Comparing PreTP-2L with the other existing methods

**Figure 5:**
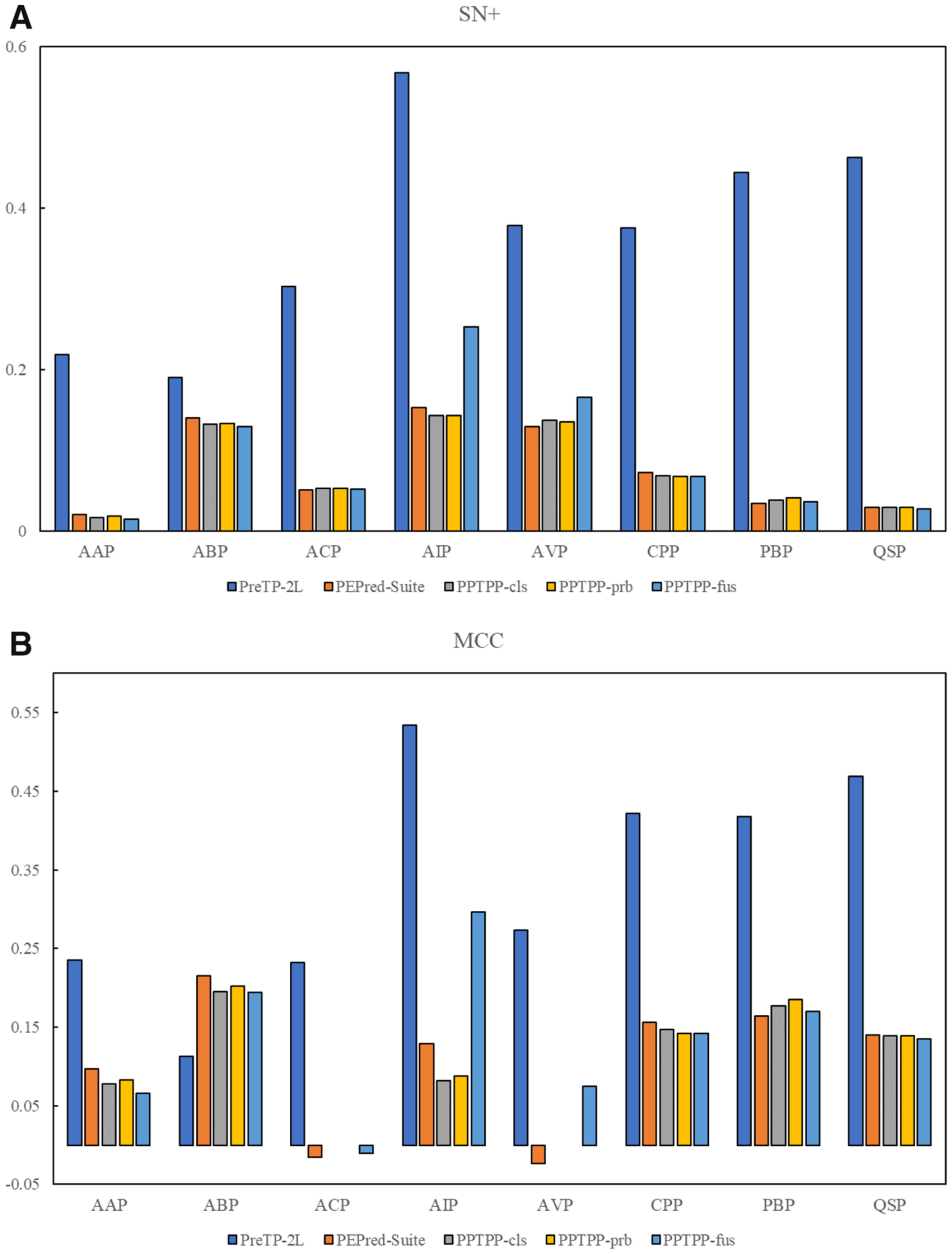
Comparison of other existing predictors on independent test dataset in terms of SN+ and MCC

We compared the prediction results of PreTP-2L with the other existing peptide prediction methods, including PEPred-Suite (Wei *et al.*, 2019), PPTPP-cls (Zhang and Zou, 2020), PPTPP-prb (Zhang and Zou, 2020), PPTPP-fus (Zhang and Zou, 2020), CAMP-SVM (Waghu and Idicula-Thomas, 2020), CAMP-RF (Waghu and Idicula-Thomas, 2020), and CAMP-DA (Waghu and Idicula-Thomas, 2020). Figure 5 and Tables 6–13 show the performance of different methods, from which we can see that PreTP-2L achieves better performance compared with the other predictors, indicating that PreTP-2L is a useful predictor for predicting the therapeutic peptides and their types. Especially, PreTP-2L outperforms the other state-of-the-art methods in terms of SN+. The results show that the proposed method can predict the fewest false positive samples than the other state-of-the-art methods, indicating that PreTP-2L is able to capture the discrepancy information between the multi-functional therapeutic peptides.

**Table 6.** Results of PreTP-2L and the other predictors for predicting the AAP on independent test dataset.

	ACC	SN+	SP	MCC
PEPred-Suite	0.573	0.020	0.570	0.097
PPTPP-cls	0.703	0.017	0.517	0.078
PPTPP-prb	0.634	0.018	0.503	0.083
PPTPP-fus	0.582	0.015	0.454	0.066
PreTP-2L	0.984	0.219	0.991	0.235

**Table 7.** Results of PreTP-2L and the other predictors for predicting the ABP on independent test dataset.

	ACC	SN+	SP	MCC
PEPred-Suite	0.554	0.140	0.527	0.215
PPTPP-cls	0.588	0.132	0.494	0.195
PPTPP-prb	0.607	0.133	0.488	0.202
PPTPP-fus	0.566	0.129	0.473	0.194
PreTP-2L	0.871	0.190	0.933	0.113

**Table 8.** Results of PreTP-2L and the other predictors for predicting the ACP on independent test dataset.

	ACC	SN+	SP	MCC
PEPred-Suite	0.299	0.051	0.276	−0.015
PPTPP-cls	0.441	0.053	0.326	0.00
PPTPP-prb	0.479	0.053	0.322	0.00
PPTPP-fus	0.485	0.052	0.292	−0.01
PreTP-2L	0.925	0.303	0.962	0.232

**Table 9.** Results of PreTP-2L and the other predictors for predicting the AIP on independent test dataset.

	ACC	SN+	SP	MCC
PEPred-Suite	0.349	0.153	0.268	0.129
PPTPP-cls	0.448	0.143	0.238	0.082
PPTPP-prb	0.735	0.143	0.226	0.088
PPTPP-fus	0.777	0.253	0.669	0.296
PreTP-2L	0.890	0.568	0.925	0.534

**Table 10.** Results of PreTP-2L and the other predictors for predicting the AVP on independent test dataset.

	ACC	SN+	SP	MCC
PEPred-Suite	0.530	0.129	0.548	−0.024
PPTPP-cls	0.523	0.137	0.623	0.00
PPTPP-prb	0.525	0.135	0.624	0.00
PPTPP-fus	0.498	0.166	0.555	0.0775
PreTP-2L	0.826	0.376	0.899	0.273

**Table 11.** Results of PreTP-2L and the other predictors for predicting the CPP on independent test dataset.

	ACC	SN+	SP	MCC
PEPred-Suite	0.485	0.072	0.465	0.156
PPTPP-cls	0.594	0.069	0.45	0.147
PPTPP-prb	0.529	0.068	0.428	0.142
PPTPP-fus	0.593	0.068	0.427	0.142
PreTP-2L	0.943	0.388	0.962	0.422

**Table 12.** Results of PreTP-2L and the other predictors for predicting the PBP on independent test dataset.

	ACC	SN+	SP	MCC
PEPred-Suite	0.792	0.034	0.790	0.164
PPTPP-cls	0.566	0.038	0.813	0.177
PPTPP-prb	0.646	0.041	0.828	0.185
PPTPP-fus	0.651	0.036	0.802	0.170
PreTP-2L	0.992	0.444	0.996	0.418

**Table 13.** Results of PreTP-2L and the other predictors for predicting the QSP on independent test dataset.

	ACC	SN+	SP	MCC
PEPred-Suite	0.679	0.029	0.676	0.140
PPTPP-cls	0.622	0.029	0.671	0.139
PPTPP-prb	0.569	0.029	0.674	0.139
PPTPP-fus	0.647	0.027	0.658	0.135
PreTP-2L	0.992	0.463	0.993	0.469

Furthermore, we also compare PreTP-2L with CAMP-SVM (Waghu and Idicula-Thomas, 2020), CAMP-RF (Waghu and Idicula-Thomas, 2020), and CAMP-DA (Waghu and Idicula-Thomas, 2020) on AMP dataset. As we can see from Table 14, PreTP-2L is able to more accurately predict AMPs, further confirming its better performance.

**Table 14.** Results of different methods on AMP datasets.

	ACC	SN+	SP	MCC
CAMP-SVM	0.557	0.215	0.538	0.143
CAMP-RF	0.555	0.217	0.533	0.149
CAMP-DA	0.549	0.212	0.530	0.136
PreTP-2L	0.766	0.283	0.859	0.146

## 4 Conclusion

In this study, we construct a new predictor called PreTP-2L to accurately predict the therapeutic peptides and their types. We construct the two-layer framework based on the CNN and VGG13 deep-learning methods to predict the specific types of the therapeutic peptides. The web server of PreTP-2L is constructed (http://bliulab.net/PreTP-2L). The users only need to input the peptide sequences, and then the therapeutic peptides and their types can be easily predicted with the help of this web server, which would be very useful for the researchers who are working on the related fields. It can be anticipated that the ensemble learning framework will have many potential applications in bioinformatics.

## Supplementary Material

btad125_Supplementary_Data

## Data Availability

The benchmark dataset and independent test dataset can be availabled in the http://bliulab.net/PreT P-2L/data/.
