# Supplementary material for: PreTP-2L: identification of therapeutic peptides and their types using two-layer ensemble learning framework
Source: Bioinformatics. 2023 Apr 3;39(4):btad125. doi: 10.1093/bioinformatics/btad125 (PMC10076046; doi:10.1093/bioinformatics/btad125)
Supplement: btad125_Supplementary_Data [file btad125_supplementary_data.docx]

**Supplementary file.**

**Table S1.** The performance of VGG13 with different parameters.

| Models | VGG13 | VGG13 | VGG13 | VGG13 |
| --- | --- | --- | --- | --- |
| Batch size | 32 | 8 | 32 | 32 |
| Epochs | 500 | 500 | 300 | 500 |
| Optimizer | Adadelta | Adadelta | Adadelta | SGD |
| ACC | 0.45 | 0.41 | 0.37 | 0.38 |
